# Prenatal Zidovudine Treatment Modifies Early Development of Rat Osteoid – Confocal Microspectroscopy Analysis

**DOI:** 10.1007/s10895-019-02429-6

**Published:** 2019-10-16

**Authors:** Zofia Drzazga, Wojciech Ciszek, Mariusz Binek

**Affiliations:** Department of Medical Physics, A. Chełkowski Institute of Physics, University of Silesia, ul. 75 Pułku Piechoty 1A, 41-500 Chorzów, Poland

**Keywords:** ECM, Collagen cross-links, Newborn rats, *Zidovudine*, Fluorescence confocal laser scanning microscopy

## Abstract

Autofluorescence of the bone extracellular matrix (ECM) has not been widely explored although the ECM plays a very important role in bone development. In our research we focused on examining the bone matrix of very young animals due to the intense growth process during the first month of life. Structure images and fluorescence spectra of the bone surface were carried out using confocal fluorescence microscope Eclipse Ti-S inverted CLSM (NIKON, Japan) for compact tibia of healthy 7-, 14- and 28-day-old rat newborns after prenatal *zidovudine* administration in comparison with control. Spectral features of ECM autofluorescence were analyzed statistically by taking into consideration *p* < 0.05. The CLSM technique allows for simultaneous examination of the structure and autofluorescence from selected areas of the bone surface. Excessive autofluorescence of ECM after prenatal zidovudine administration influences bone growth incommensurably to the newborns’ age. Therefore the possibility of an additional non-enzymatic mechanism of collagen cross-linking in the first two weeks of life of newborn rats prenatally treated with zidovudine has been considered. Our results suggest that ECM autofluorescence can be an indicator of bone development in the normal and pathological state.

## Introduction

Bone is a complex tissue whose proper functioning is based on cooperation between living cells (osteoblasts, osteoclasts, and osteocytes) and extracellular matrix (ECM) composed of organic matter dominated by collagen proteins and the inorganic component consisting mainly of hydroxyapatite. Nowadays anatomy and physiology of bone system and its cells are well described. Recently normal osteogenesis and bone development during whole human or mammalian life as well as mechanisms of disorders detected in bone physiology and bone growth have been researched by many authors, i.e. [1–6].

Our earlier work on bone focused on studies of the effect of antiretroviral drugs, such as *zidovudine* or *indinavir,* that are used among others to treat the HIV infections, on bone tissue development in an animal model [7–9]. It was shown using the XRF technique that maternal administration of *indinavir* disturbs concentration of elements of inorganic matter, especially in trace elements such as zinc and strontium whose content changes regulate the Ca level. On the other hand, prenatal *zidovudine* treatment influenced organic matter to a greater extent, which we revealed by using laser fiber spectroscopy. However, it is interesting to deepen investigation of ECM using confocal laser scanning microscope with the option of obtaining spectra from a specific microscopic area.

Advanced microscopic techniques including multi-photon microscopy are often applied in characterization of biological tissues including bone in biomedical research of oncological as well as non-oncological cases associated with environmental stress, genetic manipulation and drug treatment [10–13]. CLSM has been an effective tool for investigating bone calcification [14] as well as the cells embedded within the mineralized bone matrix. Bone-resorbing osteoclasts can be analyzed by confocal microscopy [15]. Combined CLSM and Differential Interference Contrast (DIC) microscopy methods were used for histotomography and 3D imaging of the structure and processes of osteocytes [16]. Non-destructive complementary imaging of cancellous bone contributed to the understanding of its bimodal structure, which is important for the development of tissue engineering. Analyzes of confocal images of microcracks in relation to biomechanical properties of ageing human cortical bone were also reported [17].

This paper presents the usefulness of confocal fluorescence microscopy for testing the physiological development of the bone matrix in the first month of life and the potentially negative effect of prenatal treatment with *zidovudine*. For this purpose, confocal images have been analyzed to reveal changes caused by the anti-HIV treatment in patterns of extracellular matrix of developing bones. Moreover, we found it worthwhile to check if autofluorescence of ECM could be a marker of bone development in the normal as well as pathological state.

## Material and Methods

The bones of newborn rats in three age groups (7-, 14- and 28-day-old) received from Department of Pharmacology at the Medical University of Silesia were used for this study. The section of the rats was performed with the approval of The Local Ethics Committee for Animal Experiments in Katowice (no. 9/2008) according to the Helsinki Declaration in the framework of the grant No.2POD08530 of the Polish Ministry of Education and Science. The preparation process of bone samples was described previously [7]. Tibias from rat offspring whose mothers were administered with *0.9%* NaCl were compared to newborns’ bones whose mothers received antiretroviral agent *zidovudine* at a 200 mg/kg [9]. Such a dose was chosen on the basis of earlier reports [e.g. 18] as well as our own experience, which indicated increasing mortality of rat fetuses at higher doses. *Zidovudine ZDV* (aka. *azidothymidine*) was one of the first medications used in antiretroviral therapy (ARV) as a representative of NRTIs blocking the process of replication in infected cells [19, 20]. Unfortunately, genotoxic effects of such therapies were observed, including negative influence on bone tissue [21, 22]. Hence study of bones from very young rats (less than one month) is important due to the intense growth process in this stage of development.

Measurements were performed for equinumerous groups of bones (6 tibias each) from younger newborns 7- and 14-day-old after maternal treatment with *zidovudine* and control, *7Z, 14Z, 7C* and *14C*, respectively. For 28-day-old rats only 4 tibias were measured in both control *28C* and *zidovudine 28Z* groups. The control groups are presumed to be healthy groups of rats’ tibias, where bone development is physiologically normal.

All samples were studied using Eclipse Ti-S inverted CLSM (NIKON, Japan). The images of bone surface were registered using 20x objective lens (NA 0.45) giving final zoom equal to 200x. To provide the best visibility the confocal plane was set on the flattest surface of the sample. As an excitation source for autofluorescence study, 404 nm laser diode (Melles Griot, <500 mW) was used because such excitation wavelength was found to produce the highest contrast in detecting early lesions [10, 23]. Emission signal was detected by the photomultiplier (PMT) with spectral resolution set at 5 nm in the range of 400–700 nm. Autofluorescence spectra were collected from images of compact bone surface near diaphysis focusing on intercellular substance of bone where circular ROIs (diameter 20 ± 5 μm) were assigned within confocal plane to reduce the noise of emission signal (5–6 ROIs in each sample). Background spectrum including laser beam spectrum and PMT noise was also recorded.

All spectral measurements were formulated using Origin (OriginLab, USA) software. Originally recorded fluorescence spectra from tibia of newborns rats were normalized to the maximum of excitation peak in measured tissue and then the background signal was subtracted. Moreover, spectra were normalized to average intensity calculated in the 650–700 nm range for all spectra. Such a procedure was used earlier by Mayinger et al. [24] as optimal for investigation of biological samples whose emission signal depends on the illumination and the position of the probe. This method takes into account data averaging, normalization and subtraction. This allows us to compare the fluorescence plots and to reveal substantial differences between registered spectra.

Statistical analysis was performed using STATISTICA12 software (StatSoft, USA). Obtained results of measurements were processed to eliminate outliers (Grubbs test). Results were distributed normally according to the normality tests (Kolmogorov-Smirnov). In the statistical calculations the standard level of confidence was assumed to be *p = 0.05* for ANOVA tests to estimate the presence of statistical differences amongst analyzed groups.

## Results

### Confocal Fluorescence Microscopy Imaging

**Fig. 1:**
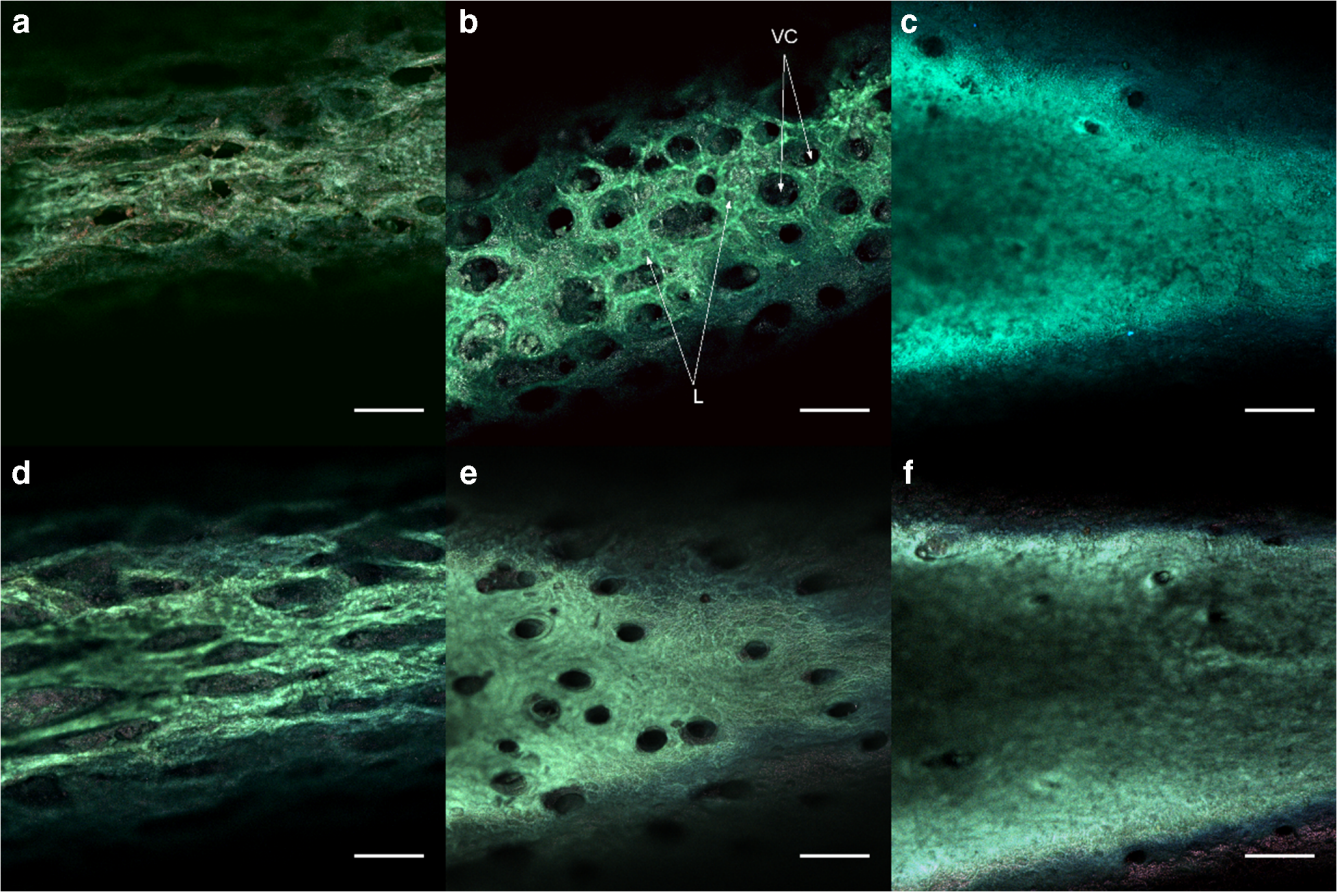
Representative age relative images of the tibia diaphysis surface of control newborn rats: **a** - *7C,***b** -*14C*, **c** - *28C*, and prenatal *zidovudine* treated newborns: **d** - *7Z*, **e** - *14Z*, **f** - *28Z*. In picture B) the Volkamnn’s channels VC and lacunae L are marked. Zoom 200x, scale bar: 100 μm

Representative images of age-related development of compact bone surface architecture within diaphysis of tibia are presented in Fig. 1. In fact, they reveal organic extracellular matrix (ECM, osteoid), composed mostly of type I collagen that gradually mineralizes to form mature bone. Collagen fibrins are the most fluorescent matter observed in the confocal plane contrasting with black areas which probably originate from the blood vessel canals (Volkmann’s canals supplying nutritional ingredients necessary for bone development) as well numerous lacunae visible in particular in the tibia of the youngest newborns.

Images registered by our microscope show eye-catching differences in size, architecture and distribution of histological elements between the studied bone groups. To assure description clarity control and *zidovudine* groups are considered separately.

Imaging revealed fragile structure of ECM in the *7C* group what is presented in Fig. 1a. Bone surface shows an irregular and spongy architecture with supramolecular structure of collagen fibrils suggesting a presence of trabecular meshwork at the beginning of bone development**.** Fig. 1b displays bone matrix patterning in 14-day-old rat bone (*14C*), altered a little due to increasing of collagen cross-linking and progressive bone mineralization. Further mineralization of proteinaceous matrix leads to the formation of dense cortical bone. Surface of the tibia from the *28C* group (Fig. 1c) demonstrates an almost mature compact bone as a result of matrix conformation from woven to lamellar bone and the process of mineralization.

Process of bone development during the first month of life of rat newborns prenatally treated with *zidovudine* is presented in Fig. 1d – f. Tibia of 7-day-old newborns (*7Z*) also show a spongy structure but self-assembly of collagen molecules into collagen fibers seems to be more advanced than in the control group (see Fig. 1d vs. a). The distinct differences in the pattern of collagen forming osteoid are observed in bones of 14-day-old newborns. Circularity of Volkmann’s canals is still noticeable but the collagen fibrils are clustered in parallel or concentric arrays characteristic for lamellar bone [1, 2]. Nevertheless, it should be noted that tibias of *zidovudine* groups are markedly bigger than the control ones in each age group.

### Autofluorescence Spectra

**Fig. 2:**
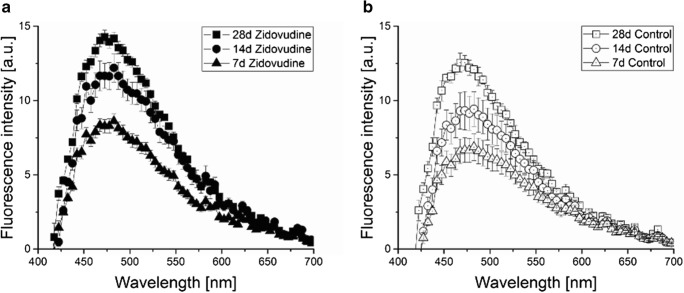
Average autofluorescence spectra of bone extracellular matrix from maternal *zidovudine* treatment group (**a)** and control (**b**) as a function of age (d – day-old) under 404 nm laser excitation

Autofluorescence spectra of the bone organic ECM measured in our experiment are presented in Fig. 2. As hydroxyapatite-like compounds are not fluorescent so the presence of it could be omitted. The characteristic broad band of emission with peak at about 480 nm is observed; apparent side band on part of longer wavelengths is found in all studied samples. Such a signal is similar to fluorescence spectra of biological tissues obtained by fiber laser spectroscopy described by various researchers earlier [9, 23, 24]. Autofluorescence in our experiment is attributed to closely spaced overlapping emissions generated by a number fluorescent compounds such as: collagen, NAD(P)H, flavins, fatty acids, vitamins and lipofuscins - see literature [25].

Emission spectra (shown in Fig. 2) disclose similar shape in all groups but the intensity of fluorescence depends on drug and age of newborns. Fluorescence normally increases with the age of newborn rats, but prenatal zidovudine drug delivery enhances the signal. The mentioned increase in the *zidovudine* group seems to be actually dependent on both ageing and drug administration processes but in different way. Therefore for better insight into the problem we analyzed: fluorescence intensity, peak position and difference in maximum between the studied groups. These basic spectral parameters allowed for a fuller description of phenomena during our study - they are listed in Table 1. For 7- and 14-day-old groups maximum peaks are located at 482.5 nm, while for the *28C* and *28Z* groups a blue-shift of maximum appears: at 15 nm and 10 nm, respectively.

**Table 1:** Mean intensity of osteoid fluorescence in maximum emission peak as a function of rat’s newborns age for control group compared with *zidovudine* group and intensity difference for successive age groups (C – control group, Z – *zidovudine* group, d – day-old)

Group	Parameters		Intensity difference between age groups Δ_intensity_		
	Maximum emission λ_max_ [nm]	Maximum intensity I_max_ [a.u.]	14d - 7d	28d - 14d	28d -7d
7C	482.5 ± 2.5	6.87 ± 0.91			
14C	482.5 ± 2.5	9.43 ± 1.17	2.56		
28C	467.5 ± 2.5	12.56 ± 0.63		3.13	5.69
7Z	482.5 ± 2.5	8.63 ± 0.51			
14Z	482.5 ± 2.5	12.20 ± 0.96	3.57		
28Z	472.5 ± 2.5	14.28 ± 0.46		2.08	5.65

Analysis of the variation of emission intensity indicates statistically significant differences (*p* < 0.001) regards age for the control bones as well as for the zidovudine group. However, no significant differences are found for groups between *14C* and *7Z* as well as between *14Z* and *28C*. This means that the fluorescence of bones of older rats from the control group is close to the emission of bones of younger rats from the *zidovudine* group. Age-dependent development rate of osteoid is estimated as a mathematical intensity subtraction Δ_intensity_ (Table 1). During the first 2 weeks of rats’ life prenatal drug administration causes faster increase of fluorescence peak intensity compared with the increase observed in the control group. However, a different effect in Δ_intensity_ was found for the next life period, between the fourth and second week of life (28d – 14d). The difference in fluorescence peak intensity is smaller in the zidovudine group than in the control group unlike the first two weeks of rats’ lives. Hence at the end of the experiment taking into account the difference between the last and first week of rats’ life (28d – 7d) – a suppression of fluorescence intensity increase between *zidovudine* and control groups was observed.

**Fig. 3:**
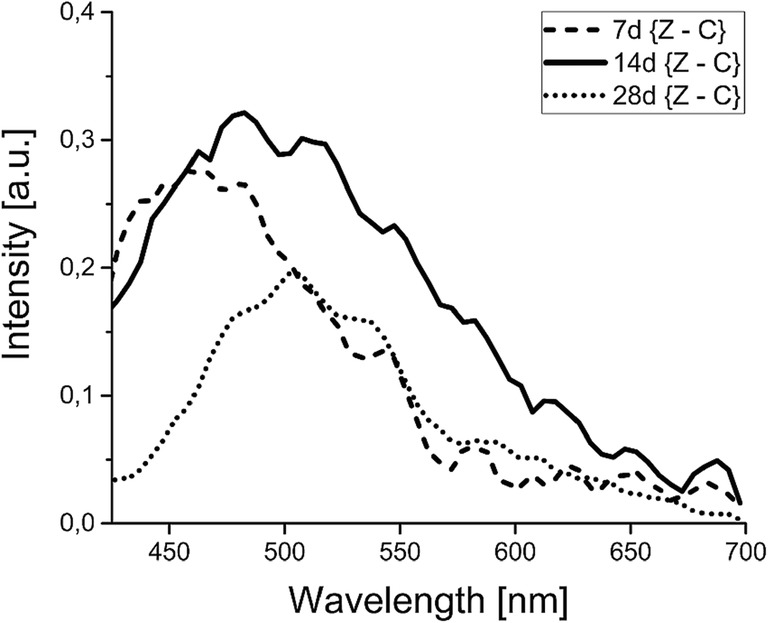
Differential plots of osteoid emission normalized to control, obtained for tibia taken from newborns after maternal treatment with *zidovudine* and control in all studied age groups (Z – *zidovudine* group, C- *control*)

Mechanisms of bone development can be better understood by using the normalized differential spectra of osteoid autofluorescence. Emission differences between the *zidovudine* group and the control group for different age of newborns is displayed in Fig. 3.

The largest excessive signal is found in bones from 14-day-old rats. The line shape reveals many component perturbations occurring through the range of the studied wave lengths. Broad signal (FWHM = 154 ± 14 nm) with maximum intensity (a.u.: 0,32 ± 0,05) located at about 482,5 nm singles out this group from among others. For 7-day-old rats a strong blue-shift maximum about 25 nm in respect to the 14-day-old group is noticeable. Differential plot of autofluorescence of tibia taken from 28-day-old rats shows the smallest excessive signal (FWHM = 96 ± 8 nm) among the studied age groups but is shifted towards red with respect to the group of 14 day old rats - located near 502,5 nm. It follows from our analysis that contributions of different endogenic fluorophores change at a varying degree with rat age.

## Discussion

In our study we focused on monitoring the development of bone matrix in newborn rats using confocal fluorescence microspectroscopy which allows to investigate selected tissue on microscopy level. The differentiated patterning of bone matrix from an initial trabecular meshwork towards dense cortical bone dependent on status bone tissue could be observed (Fig. 1). As expected, the obtained confocal images confirm that organic extracellular matrix is dominated by collagen fibrils which constitute ca. 85% of bone ECM [1]. Important aspects of alternations in bone extracellular matrix during the first month of rat newborns life are reflected in the autofluorescence measurements. It should be noted that progressive mineralization does not reduce collagen emission, as previously mentioned [26]. It is noteworthy that age and drug related changes in autofluorescence spectra of newborn rats’ bone matrix on microscope level are in a good quality agreement with results obtained from 1,8 mm diameter circular areas of compact bone by fiber laser spectroscopy [7, 9] for younger newborns.

An age-increasing autofluorescence of bone matrix seems to be primarily associated with the increasing number of fluorescent molecules, mainly of collagen ones emitting in the range 400–510 nm [25]. However, our preliminary study of autofluorescence of the surface of the compact bone by steady fluorescence spectroscopy [9] suggested essential contributions generated not only by collagen but also by nicotinamide adenine dinucleotide (NAD(P)H) in the visible range of wavelengths. Basing on autofluorescence under excitation of 404 nm obtained in this framework, it is difficult to precisely determine collagen and NAD(P)H contributions due to their overlapping emissions. In addition, the fluorescence contributions may not be additive due to complicated interactions of the fluorophores with the molecular environment. Detail analyses of collagen and (NAD(P)H), as two of the major tissue fluorescent biomarkers of pre- and cancerous changes were performed using Monte Carlo technique [27], chemometric tools [28] and MCR-ALS method [29].

Bone matrix is mostly composed of collagenous proteins but non-collagenous proteins (10 to 15% of total bone protein) including proteoglycans, glycosylated proteins, gamma–carboxylated (gla) proteins and 2HS-glycoprotein may affect bone cell activity and regulate bone mineral deposition [1]. The most prevalent non-collagenous protein - osteonectin, (approximately 2% of total protein in developing bone) is thought to affect osteoblast growth and matrix maturation regulating fibril diameter. On the other hand, calcium- and phosphate-binding proteins such as osteocalcin, osteopontin, bone sialoprotein and alkaline phosphatase participate in matrix mineralization by regulating the amount and size of hydroxyapatite crystals formed. Such an imposition of mentioned tissue processes must be taken into account during studies when prenatal treatment with antiretroviral drugs is used. It is clear that therapy in pregnancy affects the development of young bone, especially altering the activity of osteoid proteins and coenzymes. It was reported that the activity of *zidovudine* as a nucleoside analogue which competes with natural metabolites for an active place in enzymes may disturb their functions [19, 30].

The effect of maternal therapy with *zidovudine* on bone tissue formation revealed in our studies the increase of ECM emission which is in opposition to fluorescence decrease in malignant bone cells [23]. Unfortunately, in our work similar trends in ECM autofluorescence changes with respect to age and prenatal pathology (Fig. 2, Table 1) make this analysis difficult. Only normalized differential spectra enabled specification of bone tissue in relation to newborns prenatally treated with *zidovudine*. Spectral profiles of differential plots of bone ECM (Fig. 3) point to the age dependent excess of emission with the shifting of the maximum towards the shorter wavelengths for the younger rats in comparison with the oldest ones. Such behavior of the differential spectrum can be associated with the different degree of maturation of collagen fibers stimulated by altering protein growth factors as well as by possible changes in fluorescence signals of metabolic enzymes ratios, like NAD(P)H_bound/free_, and NAD(P)H_total/oxidized flavins_ on which the spectral shape can depend, as discussed in [25].

It was presented by Bailey et al. [31] that collagen cross-linking involves two different mechanisms, one enzymatically controlled cross-linking during development and maturation and the other an adventitious non-enzyme mechanism following maturation of the tissue. Advanced glycation end products (AGEs) are thought to be the major cause of the dysfunction of collagenous tissues in ageing, osteoporosis, diabetes [32] as well in cardiovascular diseases [33]. It is not excluded that an additional non-enzymatic cross-linking may have pathophysiological implications in bone ECM of rat newborns with maternal administrated zidovudine, especially during the first two weeks of rat’ life. Too rapid, non–adequate to age increase of collagen cross-linkage could be interpreted as a premature process of ageing of collagenous tissue during bone development. Taking into consideration the maximum fluorescence of AGEs about 440–450 nm occurring in biological substances, it seems likely that the abnormal increase in intensity blue-shifted fluorescence part of differential spectra is due to the additional emission of AGEs deposits in bone ECM of younger newborn rats, especially 7-day old ones. Differential spectrum of 14-day old rats is additionally influenced by fluorescence contribution covering the yellow-reddish region in which lipofuscins or lipofuscin-like lipopigments may emit (480–700 nm) [25]. The presence of these fluorophores might be a marker of the side effect of maternal treatment with drug on development of the bone matrix. Earlier physiological intracellular accumulation of lipofuscins in the central nervous system with ageing as well as in response to pathological conditions or to toxic compounds were reported [34]. Autofluorescence of lipofuscin in retinal pigment epithelium due to Stargardt’s disease and age-related macular degeneration [35, 36] was also discussed. In addition, even a small amount of lipids in the mineralized bone tissue [37] taking part in bone physiology in relation to their metabolism may influence the differential spectra (Fig. 3) at about 470–480 nm.

A reduction of side effects of prenatal drug administration was observed in the tibia of the oldest rats tested. It is noteworthy that excessive contributions at the beginning and at the end of the differentiated spectra disappear for tibia of the 28 day-old rats which might indicate a return to basic enzymatic cross-linking.

## Conclusions

Our investigations show applicability of confocal laser scanning microscopy for comprehensive characterization of organic extracellular matrix and its mineralization in the first month of life of rat newborns. Changes in the pattern of collagen forming the osteoid and differences in the emission of ECM fluorophores allow to monitor bone development in the normal and disturbed state due to prenatally administration of anti-HIV drug *zidovudine*. The possibility of an additional non-enzymatic mechanism of collagen cross-linking in the first two weeks of life of newborn rats is considered. ECM autofluorescence can be an indicator of bone development and hence may be useful in clinical trials.
